# Rapid detection of Pfcrt and Pfmdr1 mutations in *Plasmodium falciparum *isolates by FRET and *in vivo *response to chloroquine among children from Osogbo, Nigeria

**DOI:** 10.1186/1475-2875-6-41

**Published:** 2007-04-11

**Authors:** Olusola Ojurongbe, Titus O Ogungbamigbe, Adetayo F Fagbenro-Beyioku, Rolf Fendel, Peter G Kremsner, Jürgen FJ Kun

**Affiliations:** 1 Department of Parasitology, Institute of Tropical Medicine, University of Tübingen, Wilhelmstr. 27, 72074 Tübingen, Germany; 2 Department of Medical Microbiology & Parasitology College of Health Sciences, Ladoke Akintola University of Technology, Osogbo, Nigeria; 3 Department of Pharmacology & Therapeutics College of Health Sciences, Ladoke Akintola University of Technology, Osogbo, Nigeria; 4 Department of Medical Microbiology and Parasitology College of Medicine, University of Lagos, Nigeria

## Abstract

**Background:**

Chloroquine (CQ) has been in use in Africa for a long time. Because of misuse, this drug has now lost its efficacy due to the emergence of resistance strains in most parts of Africa. Recently, it was shown that after chloroquine has been withdrawn from the market, chloroquine-sensitive *Plasmodium falciparum *re-emerged and chloroquine could again be used successfully as an antimalarial. Surveillance of parasite populations is, therefore, important to decide whether chloroquine could be re-introduced.

**Methods:**

To estimate the prevalence of the most pivotal polymorphisms, including *Pfcrt *K76T, *Pfmdr1 *N86Y and *Pfmdr1 *Y184F mutations, and their contributions to the outcome of CQ treatment, isolates from Osogbo Western Nigeria were tested using the Fluorescence Resonance Energy Transfer (FRET) method on a real-time PCR instrument.

**Results:**

116 children with acute uncomplicated *P. falciparum *malaria infections were treated with the standard dosage of CQ and followed-up for 28 days. Blood samples were collected on filter paper at enrollment and during follow-up for identification of parasite carrying the chloroquine resistant transporter (*pfcrt*) and *P. falciparum*-multi drug resistance (*pfmdr1*) gene mutations. Parasitological assessment of response to treatment showed that 62% of the patients were cured and 38% failed the CQ treatment. The presence of single mutant *pfcrt *(T76) alleles (P = 0.003) and in combination with mutant *pfmdr1 *Y86 (P = 0.028) was significantly associated with *in vivo *CQR. No other mutation on its own or in combinations was significantly associated with treatment outcome. Mutant *pfcrt *was more prevalent in both pre- and post-treatment isolates. No association was observed between age or initial level of parasitaemia and chloroquine treatment outcome.

**Conclusion:**

The result established the usefulness and accuracy of real time PCR in *pfcrt *and *pfmdr1 *mutation detection and also give further evidence to the reliability of the *pfcrt *T76 point mutation as a molecular marker for CQ resistance.

## Background

While there is an active search for new antimalarial drug combinations that could prevent or delay further spread of resistance, there is a need to understand the basis of parasites resistance to chloroquine (CQ) and other antimalarial drugs and explore potentials to use the data in improving the potency and rational for selecting components for effective drug combination. Constant observation of the existing parasite population concerning their genetic makeup determining the resistance to CQ became even more important since it was shown that after CQ withdrawal for therapy CQ-sensitive parasite re-occurred [1]. So written-off drugs may come into focus again.

The molecular basis of CQ resistance in *Plasmodium falciparum *is still unclear, and the association of point mutations in different genes with chloroquine-resistance has been largely studied in the last decade. In 2000, *pfcrt *gene was identified [2]. This gene consisting of 13 exons showed 6–8 point mutations including one that appears to play a crucial role in CQR [3]. A lysine to threonine change at position 76 (K76T) which was subsequently found in every *in vitro *CQR parasite from around the world [4,5] was identified as an important mutation associated with CQR. The resistance was associated with a reduced accumulation of CQ in the parasite digestive vacuole but how the *pfcrt *gene exerts such an effect on the digestive vacuole is still unclear. Many studies have shown that the *pfcrt *play a crucial on CQR, but this mutation was not the sole requirement, suggesting that other factors including host factors are responsible for the clearance of CQR parasites [6].

Polymorphisms in *pfmdr1*, a gene located on chromosome 5 which encodes the *P. falciparum *P-glycoprotein homologue-1 is also thought to modulate CQR. It is a typical member of the ATP-binding cassette transporter superfamily localized in the parasite vacuole, where it may regulate intracellular drug concentrations [7]. Mutations were observed at the amino acids 86, 184, 1034, 1042, and 1246, which were strongly linked to the CQR in laboratory clones obtained from various regions [8]. However, the link between *pfmdr1 *and CQR still remains unclear and controversial [6,9]. While some field studies had indicated that there is positive association between CQR and mutation (asparagine to tyrosine change) at position 86 (N86Y) [10,11], several others had doubts about this association [12,13]. Currently, *pfmdr1 *mutations are said to assist the CQR parasites by augmenting the level of resistance. A combination of *pfcrt *and *pfmdr1 *polymorphisms is believed to result in higher levels of CQR [4,7].

In Nigeria, CQ has been used for many years as the first-line treatment for uncomplicated malaria. However, like many other malaria endemic regions the therapeutic efficacy of CQ has decreased considerably. This, therefore, has led to the change in the first line drug for the treatment of malaria to artemisinin-based combination, although, CQ is still widely used in the country. In order to explore the roles of *pfcrt *and *pfmdr1 *polymorphisms in CQR, the Fluorescence Resonance Energy Transfer (FRET) method was used to determine these polymorphisms and their *in vivo *sensitivity to chloroquine in *P. falciparum *isolates from Osogbo Western Nigeria. The use of a Real Time PCR assay for a rapid, sensitive, and specific detection of these mutations was also assessed.

## Materials and methods

### Study site and patients

The study was undertaken between July 2004 and January 2005 in the town of Osogbo located in the western part of Nigeria. Osogbo is the state capital of Osun state Nigeria and it represents a typical urban setting in Nigeria. Malaria is present throughout the year with a marked increase during the raining season (i.e. April – September). Febrile children (1–12 years old) attending the Osun state Hospital and Ladoke Akintola University Teaching hospital were screened for *P. falciparum *parasitaemia. Blood films were stained with 10% Giemsa and examined microscopically. Criteria for recruitment in this study were: (1) asexual parasitaemia between 2,000/μl and 200,000/μl, (2) no signs of severity or severe malaria (including severe anaemia defined by haemoglobin <5 g/dl), (3) no intake of antimalarial drugs during the preceding four weeks, (4) informed consent from the patient parent or guardian. The detected parasitaemic cases were treated with 25 mg/kg chloroquine in divided doses for three days at 10 mg/kg daily for D0 and D1 and 5 mg/kg for D2. Subsequent follow up appointments were scheduled for days 3, 7, 14, 21 and 28.

Classification of responses to treatment was done according to the WHO criteria [14]. The cure rate on day 28 of the follow-up was defined as the percentage of children who remained free of parasites. Two drops of blood were also blotted onto 3 MM Whatman filter paper on days 0 before treatment and during following up when there was re-occurrence of clinical symptoms for extraction and analysis of parasites DNA. Treatment failure rates were corrected by *msp-2 *genotyping of parasites at enrollment and recrudescence of infections [15]. The study received ethical approval from the ethical review committee boards of the joint College of Health Sciences/Ladoke Akintola University Teaching Hospital and Osun State Hospitals Management Board.

### Detection of Pfcrt and Pfmdr1 mutations by Real time PCR

Parasite genomic DNA was extracted from blood samples collected on filter paper using a QIAamp DNA blood kit (Qiagen, Hilden, Germany), according to the manufacturer's instructions. The oligonucleotide probes and primer used to detect the polymorphisms in *pfcrt *and *pfmdr1 *are shown in Table 1.

**Table 1:** Sequence of primers and probes used for *Pfcrt *and *Pfmdr1 *amplification and melting temperatures of the sensor probes of each allele

	**Melting temperatures (°C) of the sensor probes**	
**Sequence 5' to 3'**	**Wild**	**Mutant**
**Pfcrt**		
Forward Primer: CTTGTCTTGGTAAATGTGCTCA		
iLC Primer: GTTACCAATTTTGTTTAAAGTTCT
Sensor Probe: TGTGTAATTGAAACAATTTTTGCTAA	46.5 ± 0.2	65.3 ± 0.4

**Pfmdr1**		
Forward Primer: TGTATTATCAGGAGGAACATTACC		
Reverse Primer: ACCACCAAACATAAATTAACGGA		
Sensor Probe 86: ATTAATATCATCATAAATACATG	51.8 ± 0.3	56.5 ± 0.2
Anchor Probe 86: TCTTTAATATTACACCAAACACAGATAT		
Sensor Probe 184: TAAAAAATGCACGTTTGACTTTATGTATTA	53.0 ± 0.2	58.7 ± 0.3
Anchor Probe 184: CCTTTTTAGGTTTATTTATTTGGTCAT		

For *pfcrt *analysis the sensor probe labeled with fluorescein at the 3' end is designed to be perfectly complementary to the mutation site. An amplification primer iLC labeled with Cy5 on the third base from the 3'end is used as a reverse primer which is extended during amplification. During FRET, fluorescein which is excited by the light source of the Rotor Gene instrument transfers its energy to the Cy5 incorporated into the PCR product working as anchor probe [16,17]. A specific melting temperature is then obtained for each genotype: a sensor probe spanning one mismatch could still hybridize to the target sequence but will melt off at lower temperature than a sensor probe with a perfect match. Primers and probes for *pfcrt *were designed and synthesized by TIB MOLBIOL (DNA synthesis service, Berlin, Germany)

For *pfmdr1 *mutations, hybridization probes consisted of two different oligonucleotides that bind to an internal sequence amplified by forward and reverse primers Table 1. The sensor probe, labeled at the 3'end with FAM, is designed to match the mutation sites. The anchor probe, labeled at the 5' end with Cy5 and phosphorylated at the 3' end to prevent extension by Taq polymerase, is designed to conserved sequences adjacent to the mutation sites. Both probes, localised on the same DNA strand, could hybridize in a head-to tail arrangement, bringing the two fluorescent dyes into close proximity. During FRET, FAM was excited by the light source of the Rotor Gene instrument. The excitation energy was transferred to the acceptor fluorophore, Cy5, and the emitted fluorescence was measured on the Rotor Gene channel in continuous during the melting phase. A specific melting temperature is then obtained for each genotype as described above. *Pfmdr1 *primers and probes were synthesized by Operon Biotechnology (Cologne, Germany).

### PCR amplification

Amplification was performed with Rotor Gene 3000 (Corbbett, Sydney, Australia). For pfcrt forward primer was added at a final concentration of 0.4 μM, iLC primer 0.5 μM and probe at 0.2 μM. The amplification program consisted of an initial step at 95°C for 10 min, amplification was performed with 40 cycles of denaturation (95°C for 10 s), annealing (50°C for 15 s), and extension (65°C for 15 s). The melting curve program consisted of one cycle of 95°C for 15 s, and heating at 36°C to 75°C rising by 1°C.

For *pfmdr1*, the master mix contains a final concentration of 0.4 μM of both primers and 0.2 μM of both Anchor and sensor probes. For *pfmdr1 *codons 86 and 184, the PCR program was as follows: 5 min at 95°C, 40 cycles of 94°C for 10 s, 52°C for 30 s, and 72°C for 40 s. The melting program consisted of one cycle of 95°C for 15 s and heating from 36 to 85°C rising by 1°C.

### Statistical analysis

For analysis purposes, each isolate was classified based on the presence or absence of a resistance-associated allele and infections with mixed wild-type/mutant alleles of *pfcrt *or *pfmdr1 *were treated as mutants. Data were analysed using the statistical programs JMP for Windows. For univariate analysis, frequencies were compared using the Fisher's exact tests. Two sided *P *values < 0.05 indicated statistical significance. McNemar's test was used to compare the samples before and after treatment.

## Results

### Patient treatment outcome

The potential for detection of a point mutation in *pfcrt *and *pfmdr1 *genes using hybridization probes on Rotor Gene technology has been evaluated. DNA from 116 samples of patients treated with chloroquine and successfully followed up for 28 days were evaluated for *pfcrt *and *Pfmdr1 *(86 and 184) mutations.

Of these, 69 (59%) were males and 47 (41%) were females. The mean age of the children was 46 ± 36 months (6–144 months). Geometric mean parasite density was 9,061 parasites/μl of blood on the day of enrollment. While infections in 72 (62%) of the patients were cured with a standard dosage of CQ, 44 (38%) failed to respond to treatment. The clinical data and therapeutic responses of the patients stratified by age is shown in Table 2.

**Table 2:** Enrollment clinical data and therapeutic responses of patients with acute uncomplicated *Plasmodium falciparum *treated with chloroquine stratified by age

**Age **	**Freq**	**Sex**		**Temp **	**Mean **	**Mean **	**Mean **	**T76 **	**Y86 **	**F184 **	**CQ Treatment **	
**(Yrs)**		**M**	**F**	**(SD)**	**PD**	**FC**	**PC**	**(%)**	**(%)**	**(%)**	**Cured**	**Failed**
0<4	72	39	33	37.7 (± 1.13)	8261.066	2.7	2.6	67 (93%)	27 (38%)	49 (68%)	46 (58%)	26 (36%)
4<8	27	20	7	38.0 (± 1.3)	11632.6	1.2	2.4	22 (81%)	9 (33%)	20 (74%)	17 (63%)	10 (37%)
8–12	17	10	7	37.8 (± 0.84)	9015.282	1.3	2.5	16 (94%)	6 (35%)	12 (71%)	9 (53%)	8 (47%)
**Total**	**116**	**69**	**47**	**37.8 (± 1.3)**	**9061.375**	**1.7**	**2.5**	**102 (88%)**	**42 (36%)**	**81 (70%)**	**72 (62%)**	**44 (38%)**

Key: Freq = Frequency

SD = Standard deviation

PD = Parasite density on Day 0

FC = Fever clearance time in days

PC = Parasite clearance time in days

### Real-time PCR for *Pfcrt *and *Pfmdr1 *gene detection in blood samples

**Figure 1:**
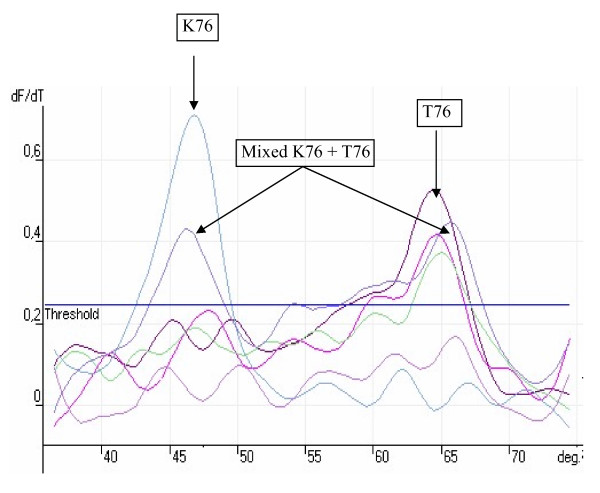
Example of a typical real time rum showing melting curve analysis of Pfcrt position 76.

**Figure 2:**
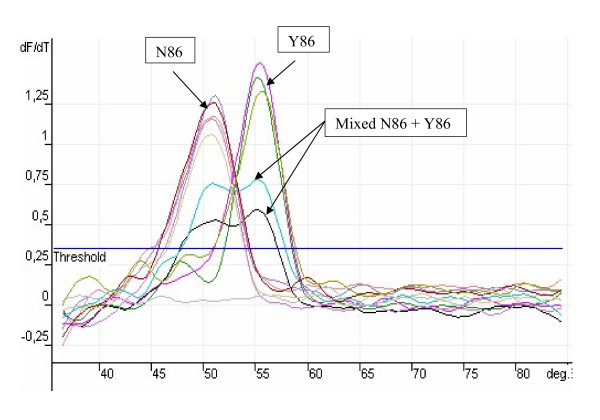
Example of a typical melting curve analysis generated by real time PCR of the Pfmdr position 86.

Wild-type and mutant alleles were rapidly determined by comparing the melting temperature for the alleles with the melting temperature of the reference alleles obtained by the FRET assay. The reference strains used for this study were 3D7, FCR, S007 and K1 laboratory strains which were all sequenced to determine the mutations that were present. A confirmed field sample of *Plasmodium malariae *was used as control along with no template. *P. falciparum *strain K1 parasites were kindly provided by Katja Becker (Justus-Liebig-University Giessen). Both 3D7 and FCR showed the wild type sequence for *pfcrt *(K76) and *Pfmdr1 *(N86 and Y184). K1 showed mutant nucleotide at position 76 (T76) for *Pfcrt *and position 86 (Y86) for *pfmdr1 *while S007 showed mutation at positions 76 for *pfcrt*, 86 and 184 for *pfmdr1*. DNA yielded a specific melting temperature of 46.5 ± 0.3°C for the *pfcrt *mutant alleles and 65.3 ± 0.4 for the wild alleles. The melting temperatures for all the alleles are shown in Table 1. The melting analysis is shown in Figures 1&2

### Association between Pfcrt and Pfmdr1 mutations and chloroquine treatment failure

The prevalence of *Pfcrt *T76 and *Pfmdr1 *(Y86 and F184) mutations known to be involved in CQR resistance in West Africa was evaluated. By *in-vivo *testing, 38% of the *P. falciparum *isolates were chloroquine resistant, although by molecular analysis 74% of the pre-treatment isolates carried the mutant *pfcrt *T76 and 14% had mixed (T76+K76) allele that has been associated with chloroquine resistance in other studies [6,18]. The *pfmdr1 *Y86 and F184 alleles were present in 29% and 64% of the pre-treatment isolates, respectively (Table 3).

**Table 3:** Prevalence of pfcrt and pfmdr1 alleles in post-treatment samples of Nigerian children with acute uncomplicated malaria.

Allele	Frequency	Treatment outcome (%)		
		Cured N = 72	Failed N = 44	P. value

N = 116				
Pfcrt T 76	86 (74%)	51 (71%)	41 (93%)	
Pfcrt K 76	11 (12%)	11 (15%)	0 (0%)	0.003*
T76+K76	19 (14%)	10 (14%)	9 (20%)	

N = 116				
Pfmdr1 Y86	34 (29%)	22 (31%)	12 (27%)	
Pfmdr1 N86	74 (64%)	47 (65%)	27 (61%)	n.s.
Y86+N86	8 (7%)	3 (4%)	5 (11%)	

N = 116				
Pfmdr1 F184	74 (64%)	45 (63%)	29(65%)	
Pfmdr1 Y 184	35 (30%)	24 (33%)	11 (25%)	n.s.
F184+Y184	7 (6%)	3 (4%)	4 (9%)	

N = 116				
T76 + Y86	37 (32%)	21 (29%)	16 (36%)	
K76 + N86	7 (6%)	7 (10%)	0 (0%)	0.028*
Mixed	72 (62%)	44 (61%)	28 (64%)	

N = 116				
T76 + F186	73 (63%)	40 (56%)	33 (75%)	
K76 + Y184	2 (2%)	2 (3%)	0 (0%)	n.s.
Mixed	41 (35%)	30 (42%)	11 (25%)	

N = 116				
T76+Y86+F184	35 (30%)	19 (26%)	16 (36%)	
K76+N86+Y184	3 (3%)	3 (4%)	0 (0%)	n.s.
Mixed	78 (67%)	50 (70%)	28 (64%)	

n.s. is not significant

The hypothesis whether allelic variations in *pfcrt *and *pfmdr1 *of *P. falciparum *isolates are associated with CQ treatment outcome was tested in the study area. The presence of *pfcrt *or *pfmdr1 *mutations or the combination of these mutations in samples collected before chloroquine treatment was examined for their association with patient treatment outcome. The *pfcrt *K76T allele was significantly associated with CQ treatment failure (P = 0. 003). Both *pfmdr1 *N86Y and Y184F did not show any significant association. The combination of *pfcrt *76 and *pfmdr1 *86 also produces weak significant association (p = 0.028) (Table 3).

For analysis of parasites strains before and after treatment, 44 pairs was collected; each consisting of the parasite strain before treatment and that from the recrudescence infection. All the 44 samples had either mutant or mixed T76 allele in their pre-treatment sample and 43/44 (98%) had the mutant *pfcrt *allele after treatment. Only one isolate showed the wild *pfcrt *allele and genotyping by MSP2 confirmed the isolate to be a new infection. For Pfmdr1 Y86 and F184 the prevalence of the alleles at pre-treatment isolates were 17% and 33%, respectively, and at recrudescence the prevalence of Y86 was 18% while that of F184 was 27%. The analysis of nonresponding and recrudescent parasites showed a pronounced significant difference between the samples collected before and after chemotherapy for the isolates having *pfmdr1 *F184 mutation (p = 0.031) while for other alleles tested there was no significant difference between the pre and post treatment samples Table 4.

**Table 4:** Prevalence of mutant *pfmdr1*/*pfcrt *genes in Day 0 and recrudescence samples of patients that failed chloroquine treatment

	Prevalence of alleles		
Alleles	Day 0	Recrudescence	Mc Nemar P value

T76	44/44 (100%)	43/44 (98%)	1
Y86	17/44 (39%)	18/44 (41%)	1
F184	33/44 (75%)	27/44 (61%)	0.031*
T76+Y86	44/44 (100%)	43/44 (98%)	1
T76+F184	44/44 (100%)	43/44 (98%)	1
T76+Y86+F184	44/44 (100%)	44/44 (100%)	1

Mixed infection is considered as mutant

*Significant at P < 0.05

## Discussion

In this study, a Real Time PCR assay for the detection of *pfcrt *and *pfmdr1 *alleles thought to be associated with CQ susceptibility and resistance was described. This Real-Time PCR assay was demonstrated to be rapid, sensitive, and specific for the detection and characterization of *P. falciparum *genetic marker of CQR. The assay detected mixed alleles infections and clearly discriminated between CQ-susceptible and CQ-resistant isolates. Its speed (up to 72 samples can be assayed in a 2-h experiment) and performance characteristics may eliminate the need for more complicated approaches and make it an attractive strategy that could easily be adapted to large-scale studies of drug resistance.

Point mutations in the *pfcrt *gene and to a lesser extent, in the *pfmdr1 *gene are thought to be associated with CQR [2,6]. The goal of this study was to evaluate the utility of these molecular markers as indicators of chloroquine resistance in isolates of *P. falciparum *obtained from Osogbo western Nigeria using a hybridization probe method on a Real Time PCR technology platform. The result of this study showed a high prevalence of *pfcrt *T76 (74%). This observation is consistent with the previous reports from various malaria endemic regions where chloroquine has been widely used. A significant association was also found between the overall *in vivo *rate of treatment failure and the frequency of mutated *pfcrt *gene in the population (P = 0.004) as already shown previously in western Nigeria [19] and other malaria endemic areas [18]. 93% of the pre-treatment isolates carried the *pfcrt *T76 and mixed allele while among the post treatment isolate the prevalence was 98%. The facts that this polymorphism was present in all the recrudescence isolates emphasised again the fact that this mutation is important in CQR.

The point mutation of asparagine to tyrosine at codon 86 of *pfmdr1 *has been associated with CQR in some studies [8,9] but not in others [12]. In the present study, both the *pfmdr1 *Y86 and F184 mutations showed no correlation with resistance to chloroquine. Both the wild type and the mutant alleles for each locus were present in both sensitive and resistance isolates. Previously, an association was established between chloroquine-resistance and alleles of the *pfmdr1 *gene in laboratory isolates obtained from different parts of the world [8]. Others had considered whether this association existed in parasite isolates obtained directly from the field [9,20]. Their analysis showed that African isolates predominantly possess polymorphism at two alleles, codon-86 and codon-184, with a positive association, although incomplete, between mutation of codon-86 and CQR. A similar association has been found in several studies [4,11,21,22]. Nevertheless, much confusion has surrounded the association of different *pfmdr1 *alleles to chloroquine resistance because numerous studies have had contradictory results. Transfection studies as well as carefully controlled molecular epidemiologic studies have shown that there are strong associations between *pfmdr1 *polymorphisms and antimalarial resistance [7]. However, like many other studies [12] the present findings have failed to find such associations because the presence of both wild-type and mutant-type alleles in our samples were largely independent of their *in vivo *responses. Also the current belief that the combination of *pfcrt *and *pfmdr1 *polymorphisms result in higher levels of CQR [4] was not observed in this study. Although a significant association was observed (p = 0.028), it was not in any way stronger than the one observed with *pfcrt *alone. Analysis of altered gene expression and other mechanisms that may contribute to a resistant phenotype is needed before the role of *pfmdr1 *can be excluded.

A recent report from Ibadan Nigeria a neighbouring town to Osogbo had suggested an association and linkage disequilibrium between the *pfcrt *T76 and *pfmdr1 *Y86 alleles in chloroquine-resistant isolates [19]. On the contrary this study suggested no possible association between these two polymorphic alleles and *in vivo *chloroquine resistance and that these molecular markers by themselves may not predict *in vivo *chloroquine resistance.

## Conclusion

In summary, the results of this study give further evidence to the reliability of the 76T *pfcrt *point mutation as a molecular marker for CQ resistance. Conversely, the role of the Y86 and F184 point mutation of *pfmdr1 *gene in CQ resistance remains elusive. The analysis of the relevant mutations by RT-PCR provides a rapid and reliable method for epidemiological and clinical studies suited for higher throughput.

## Authors' contributions

OO performed the *in vivo *testing and the molecular analysis, and drafted the manuscript.

TOO performed the *in vivo *testing.

FR participated in the molecular study.

AFF-B supervised the design of the study.

PGK and JFJK supervised the molecular work and the interpretation of the data and helped to draft the manuscript.

All authors read and agreed to the content of the manuscript.
